# Relationships and Within-Group Differences in Physical Attributes and Golf Performance in Elite Amateur Female Players

**DOI:** 10.3390/life14060674

**Published:** 2024-05-24

**Authors:** Luke Robinson, Andrew Murray, Daniel Coughlan, Margo Mountjoy, Jack Wells, Rebecca Hembrough, Danny Glover, Fiona Scott, Anthony Turner, Chris Bishop

**Affiliations:** 1 London Sport Institute, Middlesex University, StoneX Stadium, Greenlands Lane, London NW4 1RL, UK; luker470@gmail.com (L.R.); a.n.turner@mdx.ac.uk (A.T.); 2 The R&A, St. Andrews KY16 9JD, UK; amurray@europeantourgroup.com (A.M.); dan@dancoughlan.com (D.C.); 3 European Tour Group, Wentworth Drive, Virginia Water, Surrey GU25 4LX, UK; 4 Ladies European Tour, Buckinghamshire Golf Club, Denham Court Drive, Uxbridge UB9 5PG, UK; dannyglover@nhs.net (D.G.); tourexercise@ladieseuropeantour.com (F.S.); 5 England Golf, National Golf Centre, The Broadway, Woodhall Spa LN10 6PU, UK; jack.wells@aru.ac.uk (J.W.); rebecca.hembrough@englandgolf.org (R.H.); 6 International Golf Federation, Maison du Sport International, Av. de Rhodanie 54, 1007 Lausanne, Switzerland; mountjm@mcmaster.ca; 7 Department of Sport and Exercise Sciences, Anglia Ruskin University, Cambridge CB1 1PT, UK

**Keywords:** women’s golf, youth athletes, strength and conditioning

## Abstract

The aim of the present study was to examine the association between a comprehensive physical testing battery and measures of golf performance in elite female amateur players. Nineteen category one (handicap ≤ 5) or better golfers (age: 16.26 ± 1.28 years, height: 166.26 ± 3.62 cm, mass: 64.04 ± 11.27 kg, wingspan: 146.53 ± 15.59 cm, handicap: +1.45 ± 0.7) volunteered to participate in this investigation. All golfers attended a single 90 min testing session where golf shot data (clubhead speed [CHS], ball speed, carry distance, and smash factor) were measured with a Trackman 4 launch monitor and a battery of physical assessments were carried out. These included anthropometric data and assessments for seated thoracic rotation, the isometric mid-thigh pull (IMTP), isometric bench press, countermovement jump (CMJ), and seated medicine ball throws for distance. Pearson’s *r* correlations showed CHS was the golf metric that most commonly demonstrated large associations with physical testing data, most notably with force at 100 ms during the isometric bench press (*r* = 0.70). Median split analysis was also conducted for the IMTP (force at 200 ms), isometric bench press (force at 100 ms), and CMJ (positive impulse). The results showed that players who produced more force at 200 ms during the IMTP exhibited a greater CHS (*g* = 1.13), ball speed (*g* = 0.90), and carry distance (*g* = 1.01). In addition, players with a greater positive impulse during the CMJ showed a greater ball speed (*g* = 0.93), carry distance (*g* = 1.29), and smash factor (*g* = 1.27). Collectively, these results highlight the relevance of explosive force production capabilities in both the lower and upper body for female golfers. This information can be used by practitioners to better target key physical attributes during testing and training of female players.

## 1. Introduction

Success in golf is determined by getting the ball into the hole in as few strokes as possible. Consistently achieving lower scores leads to a lower golfing handicap, which is indicative of a golfer’s skill level [1,2]. Within the professional game, “gross” scores are utilised, with lower scores on the golf course typically leading to higher financial reward [3]. As the game of golf has progressed, the procedures for measuring, monitoring, and improving performance have also progressed. Strokes gained is a performance analysis tool developed by Broadie [4], which measures the quality of each shot based on its starting location and finishing proximity to the hole. Previous research has highlighted that on average, a 20-yard increase in distance translates to 0.75 strokes saved per round in PGA Tour players [5]. Naturally, this type of information has placed a huge emphasis on players trying to improve technical aspects of golf; note that it is a sport heavily under-pinned by skill [6].

Traditionally, golf is not a sport with a long-standing history of strength and conditioning (S&C) training. However, recent research now indicates a growing awareness of this type of training as part of attempts to improve golf shot performance and over-arching player health [7,8,9]. For example, it is now widely accepted that improving strength and power (in both the upper and lower body) is associated with a golfer’s ability to generate increased force production, which is fundamental for achieving the maximum clubhead speed (CHS) [7,8]. Specifically, and when focusing on lower body strength, Oranchuk et al. [10] demonstrated a sizeable relationship (*r* = 0.64) between the one-repetition maximum (1 RM) back squat and CHS. Furthermore, when focusing on jump metrics such as positive impulse and peak power, a collective body of research has reported large to very large associations (*r* = 0.61–0.79) with CHS [11,12,13]. From an upper body perspective, Keogh et al. [14] and Torres-Ronda et al. [15] both found moderate to large associations between the 1 RM bench press and CHS (*r* = 0.50), peak ball speed (*r* = 0.61), and average ball speed (*r* = 0.62). Collectively, these findings emphasise the importance of strength and power in both the lower and upper body. However, it is important to note that all of the previously mentioned research is centred around male golfers, some of which may not be directly transferable to the female game [16].

Female-specific research relating to the associations between physical attributes and golf performance is available but in a much-reduced quantity. Marshall et al. [17] investigated the relationship between flexibility, balance, and CHS in collegiate golfers. The authors used the Balance Error Scoring System (BESS) to provide an assessment of balance and stability, whilst also incorporating the sit-and-reach test for flexibility. The results demonstrated that lower BESS scores had a relationship with the average distance (*r* = −0.714; *p* < 0.05), whilst greater sit-and-reach lengths had a significant relationship with the maximum driving distance (*r* = −0.722; *p* < 0.05) and maximum CHS (*r* = −0.735; *p* < 0.05). However, despite these findings, it should be noted that associative analysis was conducted with a sample size of five, which is undoubtedly too low for any meaningful interpretation of data [18] and for inferences to be made to a wider golfing population. In addition, given the rotational nature of the golf swing, there are some queries around the ecological validity of the sit-and-reach test for golfers.

More recently, Coughlan et al. [16] found significant associations between CHS and the countermovement jump and ballistic medicine ball throws in young female golfers. Specifically, positive associations were evident between CHS and CMJ power (*r* = 0.60; *p* < 0.05), a seated medicine ball throw for distance (*r* = 0.35; *p* < 0.05), and a rotational medicine ball throw for distance (*r* = 0.56–0.57; *p* < 0.05). Finally, Brown et al. [19] reported positive associations between CHS and grip strength on the left hand (in right-handed golfers) (*r* = 0.54; *p* < 0.05), and seated flexibility in both the clockwise and counter-clockwise directions (*r* = 0.52–0.71; *p* < 0.05). With only three studies available in female golfers that relate to the association between S&C training and some surrogate measure of golf performance, it is clear that further research is warranted [9]. Furthermore, with some questionable assessment methods for physical characteristics selected in previous studies with female players [17,19], there is a need to develop a more robust and comprehensive testing battery to truly elucidate the relationship between physical characteristics and measures of golf performance in female players. Consequently, this was the primary aim of the present study.

## 2. Methods

### 2.1. Study Design

The current methodology was based on a similar, recent study conducted in male golfers [20]. The participants completed all assessments in a single session, which lasted approximately 90 min. Testing took place either in a national or regional performance centre, specifically for the national governing body for golf in England. Golf performance testing took place prior to physical testing to ensure that minimal fatigue impacted data collection. Three trials were conducted for both physical testing and golf shot data in line with previous research [16], with an average of all trials used for subsequent data analysis. Prior to any data collection, a participant’s height (cm), body mass (kg) and wingspan (cm) were measured. Participants also reported their current handicaps during testing, which were corroborated by the England Golf app, where all players were registered.

### 2.2. Participants

Nineteen female category one (handicap ≤ 5) or better golfers (age: 16.26 ± 1.28 years, height: 166.26 ± 3.62 cm, mass: 64.04 ± 11.27 kg, wingspan: 146.53 ± 15.59 cm, handicap: +1.45 ± 0.7) volunteered to participate in this investigation. All participants were deemed to be high-level amateur golfers, having played competitively for a minimum of 3 years at either a regional or national level. In addition, each player had a minimum of 12 months structured S&C training experience as part of the regional or national England Golf programme. Written informed consent was gained from participants’ parents or legal guardians, in addition to subject ascent prior to testing, with ethical approval granted by the the London Sport Institute research and ethics committee at Middlesex University, London, UK.

### 2.3. Procedures

#### 2.3.1. Warm-Up

Prior to any data collection, the Head of Strength and Conditioning for England Golf (co-researcher) conducted a warm-up. This consisted of a range of dynamic flexibility exercises, performing 1 set of 12 repetitions for forward lunges, lateral lunges, push ups, and ‘the world’s greatest stretch’ [7], with the primary aim to prepare the players for the subsequent golf and physical testing [21]. Following these dynamic stretches, players were provided with the autonomy to undertake their own golf swing warm-up, noting that individual preferences are adhered to in real-life settings, a concept which has been applied in recent golf research [20,22].

#### 2.3.2. Golf Shot Data

All participants utilised their own driver and were instructed to “hit the ball as if you were on the tee box of a Par 5, with a 50-yard-wide fairway”, whilst aiming for a specific and consistent target on the driving range for each shot. Participants were able to set the tee height according to their own preferences, utilised Titleist ProV1 golf balls for each shot, and were provided with a minimum of 90 s rest between trials. All shots were captured outdoors by a TrackMan 4 launch monitor (Trackman, Vedbaek, Denmark), which is considered to be one of the gold-standard launch monitors in the field [22,23]. The metrics recorded were (i) CHS, (ii) ball speed, (iii) carry distance, and (iv) smash factor, which were selected because recent research has shown them to be reliable both within [23] and between test sessions [22].

#### 2.3.3. Seated Thoracic Rotation

The iPhone compass application was used to assess thoracic rotation, as it has been previously reported as being a reliable and valid tool to measure thoracic rotation in comparison to other current, gold-standard universal goniometers [24]. Prior to testing, participants sat on the edge of a bench, with the researcher standing behind them, ready to carry out the assessment. To ensure consistent and reliable testing protocols, participants were instructed to place their feet flat on the floor, with the height of the bench enabling a 90-degree flexion angle at the hip and knee joints. In order to minimise movement in the lower body, a second researcher observed and ensured the starting alignment of the knees and hips were maintained throughout the assessment. Further to this, participants were instructed to cross their arms over their chest, sitting as upright as possible. The iPhone was positioned between the T1 and T2 vertebrae of each participant, ensuring the iPhone read 0° on the compass app. The iPhone was securely held against the participant, while they rotated as far as possible in the chosen direction. Each participant performed rotations in both directions, followed by a consistent 30 s rest period between each trial.

#### 2.3.4. Countermovement Jump

Twin force plates (Hawkin Dynamics, Westbrook, ME, USA) were employed as an assessment tool for the CMJ. Prior to the collection of data, the researchers ensured the force platforms underwent a calibration process as per the manufacturer’s guidelines. This included ensuring the force plates were level on the ground and evaluating whether the force plates had finished booting and zeroing and entered pairing mode prior to testing. The researchers provided the participants with a demonstration of the correct CMJ technique, alongside verbal cues of “jump as high as you can” prior to the commencement of data collection. Participants were instructed to utilise a self-selected depth during the countermovement, ensuring there was no alteration to natural jumping coordination strategies. Data for the following metrics were collected: (i) jump height: calculated using the impulse-momentum method, (ii) peak propulsive power: quantified by the peak instantaneous mechanical power applied to the system centre of mass during the propulsion phase, (iii) peak propulsive force: quantified as the maximum force value prior to take-off, and (iv) positive impulse: the total vertical impulse applied to the system centre of mass during the braking and propulsion phase [12,13].

#### 2.3.5. Seated Medicine Ball Throw

Two researchers were involved in the monitoring and collection of data for this assessment. A 3 kg medicine ball, an adjustable weight bench, and measuring tape were the equipment included. Prior to data collection, the bench was set in an upright position (i.e., approximately 110 degrees), with a tape measure fixed to the floor, enabling the distance thrown to be measured from each trial. Participants were instructed to sit on the bench with their back firmly placed against the upright back rest, with their feet firmly on the floor. From this position, participants held the medicine ball at chest height, before extending their elbows as explosively as possible, to throw the medicine ball in a ‘chest pass’ style of throw as far as they could. Participants were instructed to keep constant contact between their back and the bench, with their feet remaining on the floor at all times. Participants were provided with 90 s of rest between each trial.

#### 2.3.6. Isometric Bench Press

The force plates were positioned directly underneath the bench, where the head and upper body were positioned. The researchers provided the participants with a demonstration of the correct technique, alongside verbal cues prior and throughout the assessment. Participants initially lay down on the bench (under the bar), with their forearms positioned vertically, elbows at 90° flexion, and with the bar in line with the mid-chest. Participants were instructed to keep their feet firmly on the floor, and no arching of the lower back was allowed during testing. Prior to data collection, three practice trials were provided at an escalating perceived level of effort, with participants asked to provide the maximal effort on the final practice trial. For data collection, subjects were given a 5-second count down, followed by a verbal cue to “push as hard and as fast as you can against the bar” in line with previous suggestions for multi-joint isometric tasks [25,26]. Three minutes of rest was provided between each trial, and the following metrics were collected from the force–time curve: i) peak force: the maximum force value in Newtons (N) recorded during the protocol, and ii) force at 100 ms, 200 ms, and 300 ms, which were the force values produced at each specific time point from the initiation of the push [27].

#### 2.3.7. Isometric Mid-Thigh Pull

All testing was again performed on force plates. For optimum positioning of the bar, it was initially positioned at a height that allowed the participant to mimic the start of the second pull position during the power clean [25]. The bar height was modified for individual participants to achieve optimal knee (125–145°) and hip angles (140–150°), in line with previous recommendations [28,29]. As per the isometric bench press assessment, three practice trials were provided at an escalating perceived level of effort, with participants asked to provide the maximal effort on the final practice trial. Once practice trials were completed, participants were instructed to initiate the starting position and to “pull as hard and as fast as possible”. If a countermovement was highlighted on the force–time curve by a value that exceeded 5 standard deviations (SD) of body mass, the trial was deemed invalid and repeated [30]. Three minutes of rest was provided between each trial, and similar to the isometric bench press, the peak force and force at 100 ms, 200 ms, and 300 ms were recorded.

### 2.4. Statistical Analysis

All data were recorded as means and SD in Microsoft Excel. Normality of the data was confirmed using the Shapiro–Wilk test (*p* > 0.05). To assess the within-session reliability of all test measures, a two-way random intraclass correlation coefficient (ICC) with absolute agreement and 95% confidence intervals (CI) and the standard error of the measurement (SEM) were used. Interpretation of ICC values was in accordance with previous research by Koo and Li [31], in which there are values of <0.5 = poor, 0.5–0.75 = moderate, 0.75–0.9 = good, and >0.9 = excellent. Pearson’s *r* correlation analysis was utilised to assess the magnitude of associations between golf performance metrics and physical assessment data. In line with prior research, correlations were categorised as 0–0.09 = trivial, 0.1–0.29 = small, 0.3–0.49 = moderate, 0.5–0.69 = large, 0.7–0.89 = very large, and ≥0.9 = nearly perfect [32].

**Figure 1:**
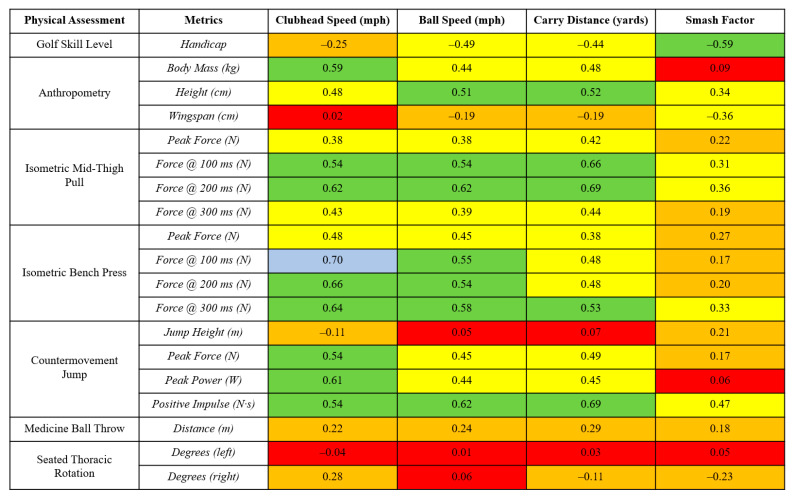
A heatmap showing Pearson’s r correlations between golf shot data (using a driver) and a physical testing battery. Note 1: Pearson’s r scale: 0–0.09 = trivial (red), 0.10–0.29 = small (orange), 0.30–0.49 = moderate (yellow), 0.50–0.69 = large (green), 0.70–0.89 = very large (blue). Note 2: kg = kilograms, cm = centimetres, N = Newtons, m = metres, W = Watts, N·s = Newton seconds.

Finally, a median split analysis was undertaken, which created higher (*n* = 10) and lower (*n* = 9) groups for the (i) isometric mid-thigh pull (IMTP) at 200 ms, (ii) isometric bench press at 100 ms, and (iii) CMJ positive impulse. Between-group differences were then assessed for each golf shot variable. Given the volume of physical capacity metrics reported in the present study, these were selected for median split analysis because they exhibited acceptable reliability and showed the strongest relationships with golf shot data (Figure 1). Due to data being normally distributed, the difference between groups was assessed using paired-samples *t*-tests, with statistical significance set at *p* < 0.05. Hedges’ *g* effect sizes (ES) with 95% CI were also used to determine the magnitude of differences between groups. These were interpreted as *g* < 0.35 = trivial; 0.35–0.80 = small; 0.81–1.50 = moderate; and >1.5 = large [33].

## 3. Results

### 3.1. Mean Data and Within-Session Reliability

The mean, SD, and within-session reliability data for driver performance and physical attributes can be seen in Table 1. For golf shot data, ICC data ranged from moderate to excellent (ICC range = 0.70–0.94), with the lowest value attributed to the ratio metric of smash factor (0.70) and the highest value for CHS (0.94). For physical attributes, ICC values also ranged from moderate to excellent (ICC range = 0.63–0.99), with the lowest value attributed to force at 100 ms during the IMTP (0.63) and the highest value for positive impulse during the CMJ (0.99).

### 3.2. Associations with Golf Shot Data

Due to the volume of correlations reported, a heatmap os presented to demonstrate the magnitude of associations between golf shot metrics and physical test scores (Figure 1). For CHS, associations with anthropometry were trivial to large (*r* = 0.02 to 0.59), and these were moderate to large for IMTP variables (*r* = 0.38 to 0.62), moderate to very large for isometric bench press variables (*r* = 0.48 to 0.70), trivial to large for CMJ variables (*r* = −0.11 to 0.61), small for the medicine ball throw distance (*r* = 0.22), and trivial to small for thoracic rotation (*r* = −0.04 to 0.28). For ball speed, the relationships with anthropometry were small to large (*r* = −0.19 to 0.51), and they were moderate to large for IMTP variables (*r* = 0.38 to 0.62), moderate to large for isometric bench press variables (*r* = 0.45 to 0.58), trivial to large for CMJ variables (*r* = 0.05 to 0.62), small for the medicine ball throw distance (*r* = 0.24), and trivial (*r* = 0.01 to 0.06) for thoracic rotation. For the carry distance, correlations with anthropometry were trivial to large (*r* = −0.19 to 0.52), and they were moderate to large for IMTP variables (*r* = 0.42 to 0.69), moderate to large for isometric bench press variables (*r* = 0.38 to 0.53), trivial to large for CMJ variables (*r* = 0.07 to 0.69), small for the medicine ball throw distance (*r* = 0.29), and trivial to small for thoracic rotation (*r* = 0.03 to −0.11). For the smash factor, the relationships with anthropometry were trivial to moderate (*r* = 0.09 to −0.36), and they were small to moderate for IMTP variables (*r* = 0.19 to 0.36), small to moderate for isometric bench press variables (*r* = 0.17 to 0.33), trivial to moderate for CMJ variables (*r* = 0.06 to 0.47), small for the medicine ball throw distance (*r* = 0.18), and trivial to small for thoracic rotation (*r* = 0.05 to −0.23).

### 3.3. Median Split Analysis

**Figure 2:**
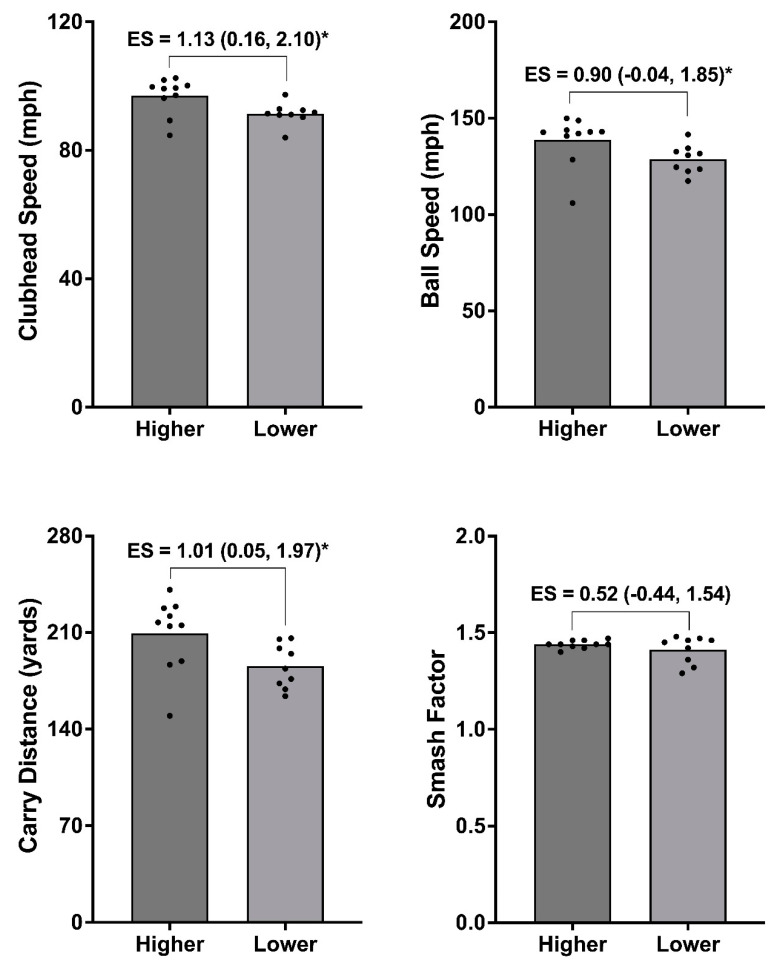
Median split analysis showing higher (*n* = 10) vs. lower (*n* = 9) groups for force at 200 ms during the isometric mid-thigh pull, and subsequent differences in golf shot data when using a driver. * Significant difference between groups (*p* < 0.05) as shown by Hedges’ *g* effect size (ES) data with 95% confidence intervals.

**Figure 3:**
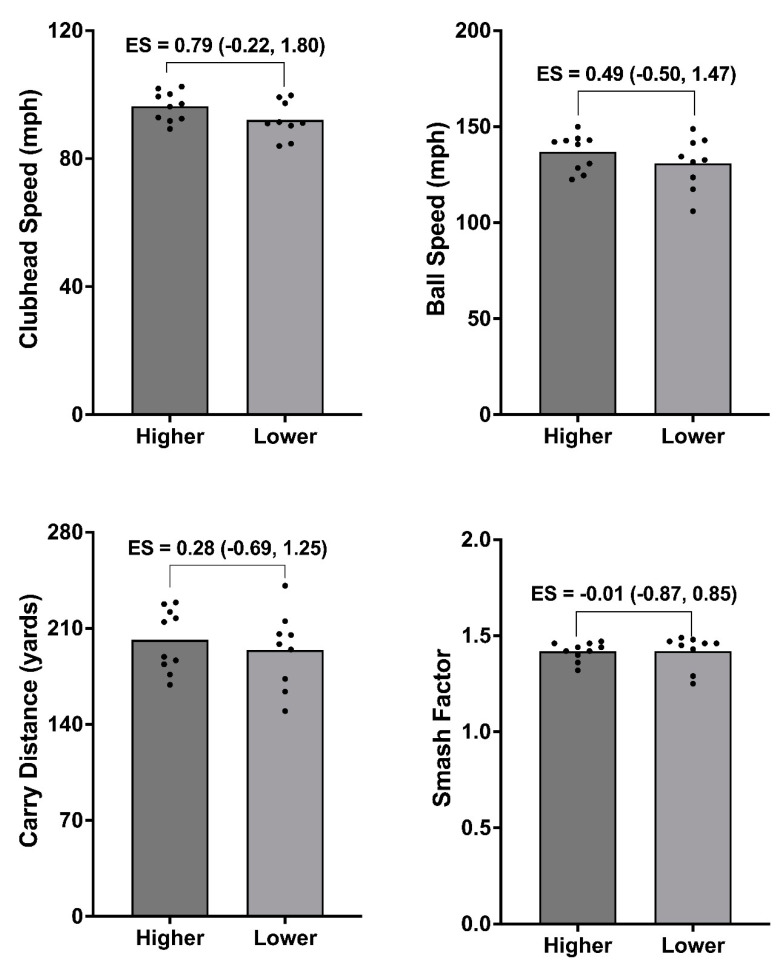
Median split analysis showing higher (*n* = 10) vs. lower (*n* = 9) groups for force at 100 ms during the isometric bench press and subsequent differences in golf shot data when using a driver. Differences between groups are shown by Hedges’ *g* effect size (ES) data with 95% confidence intervals.

**Figure 4:**
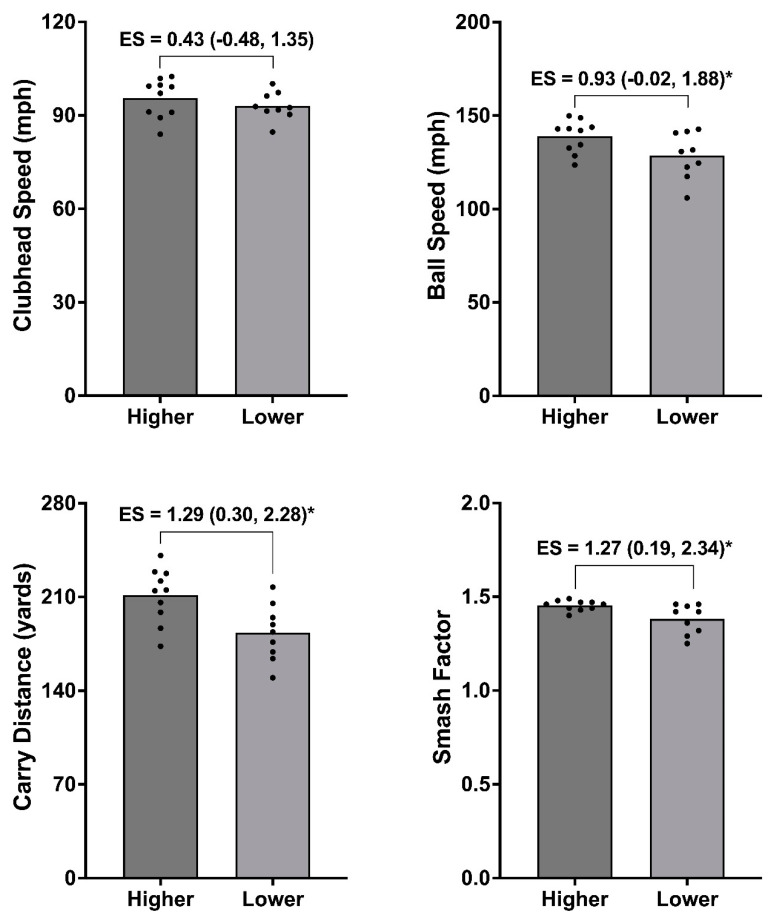
Median split analysis showing higher (*n* = 10) vs. lower (*n* = 9) groups for CMJ positive impulse and subsequent differences in golf shot data when using a driver. * Significant difference between groups (*p* < 0.05) as shown by Hedges’ *g* effect size (ES) data with 95% confidence intervals.

Figure 2, Figure 3 and Figure 4 show the median split analysis for the IMTP force at 200 ms (Figure 2), isometric bench press force at 100 ms (Figure 3), and CMJ positive impulse (Figure 4). Once these splits were made, a comparison of the golf shot data was carried out for all golf shot metrics (CHS, ball speed, carry distance, and smash factor). Figure 2 (IMTP force at 200 ms) shows moderate differences between the groups for CHS (*g* = 1.13 [0.16, 2.10]; *p* < 0.05), ball speed (*g* = 0.90 [–0.04, 1.85]; *p* < 0.05), and carry distance (*g* = 1.01 [0.05, 1.97]; *p* < 0.05), and small, non-significant differences for the smash factor (*g* = 0.52 [−0.44, 1.54]). Figure 3 (isometric bench press force at 100 ms) shows non-significant differences between groups for all golf shot measures. Specifically, moderate differences were evident for CHS (*g* = 0.79 [–0.22, 1.80]), small differences in ball speed (*g* = 0.49 [–0.50, 1.47]), and trivial differences for the carry distance (*g* = 0.28 [–0.69, 1.25]) and smash factor (*g* = –0.01 [–0.87, 0.85]). Finally, Figure 4 (CMJ positive impulse) also shows small, non-significant between-group differences for CHS (*g* = 0.43 [–0.48, 1.35]); however, statistical significance are evident between groups for the ball speed (*g* = 0.93 [–0.02, 1.88]; *p* < 0.05), carry distance (*g* = 1.29 [0.30, 2.28]; *p* < 0.05), and smash factor (*g* = 1.27 [0.19, 2.34]).

## 4. Discussion

The aim of the present study was to examine the association between a comprehensive physical testing battery and measures of golf performance in elite female amateur players. The results showed trivial to very large associations between physical attributes and golf shot data. Specifically, CHS exhibited the strongest and most consistent relationships of all golf shot measures, with the isometric bench press assessment the only protocol exhibiting very large correlations (Pearson’s *r* for force at 100 ms = 0.70). Median split analysis was also conducted, with stronger players from the IMTP (force at 100 ms) exhibiting a significantly greater CHS (*g* = 1.13), ball speed (*g* = 0.90), and carry distance (*g* = 1.01). Further to this, players exhibiting a larger positive impulse during the CMJ showed a significantly greater ball speed (*g* = 0.93), carry distance (*g* = 1.29), and smash factor (*g* = 1.27).

### 4.1. Associations with Golf Shot Data

Figure 1 shows a heatmap providing Pearson’s *r* correlations between physical assessments and measures of golf shot performance. Firstly, it is important to acknowledge that for single-time-point data such as this, we are likely only concerned with any relationships that are large (i.e., greater than 0.5). With this in mind, CHS showed the most meaningful relationships (i.e., greater than 0.5), with one very large (blue) and eight large (green) associations. Ball speed showed seven large associations with physical testing, carry distance showed five, and the smash factor only one—but this was for the handicap, which is not an assessment method for physical capacity. Thus, with both CHS and ball speed showing stronger associations with the physical performance data, they appear to be two of the more important golf proxy measures, especially when compared to the smash factor, a notion that has been discussed in recent golf studies [20,22].

When focusing on specific physical attributes, rapid force production during the isometric strength assessments (i.e., force at 100 ms, 200 ms, and 300 ms) exhibited notably stronger associations than peak force, especially for CHS and ball speed, which may be explained by one logical interpretation. Firstly, the literature has outlined that from address to impact, the duration of the golf swing is approximately 1 s [34]. However, with the proximal to distal sequencing that occurs throughout the kinetic chain during the swing, it has been hypothesised that golfers may only have approximately 0.3 s to ball impact from the moment that their centre of pressure begins to shift towards the target line, just prior to the downswing visibly starting [35]. With this in mind, previous research has shown that it may take a minimum of 0.4 s for maximal force production to be reached [36]. Thus, it seems plausible to suggest that golfers do not have sufficient time during the swing to generate maximal force. Rather, their ability to produce force quickly may be more important and better aligns with the high-velocity nature of the golf swing, which, in turn, will also have a knock-on effect on increased ball speed. This logical reasoning may help to explain why similar associations were seen for both CHS and ball speed.

Continuing with our associative findings, the jump height from the CMJ appears to be of little relevance to these golf proxy measures. This may be, in part, due to the relationship with body mass (which showed strong relationships with CHS–see Figure 1). Put simply, additional body mass may be useful for helping to swing the club faster, but it may potentially hinder an athlete’s ability to jump higher, which was suggested in a recent meta-analysis examining the link between physical characteristics and CHS in golf [35]. Further to this, our findings indicate that a better metric from the CMJ might be the positive impulse, which collectively showed strong associations with CHS, ball speed, and carry distance. This is in full agreement with the aforementioned meta-analysis (albeit this was in male players), which showed the jump impulse to have the largest summary effect estimated on CHS [12,13,35]. This may be because the jump height is a crude outcome measure of the jump performance, whereas metrics such as impulse provide a representation of how explosive force is produced over time, a concept that is critical during the golf swing as well.

Our data also show that measures of mobility do not appear to be strongly associated with golf shot data. Historically, golf has been a sport where a strong belief has been held that players need to be mobile and flexible in order to perform the golf swing well. However, recent evidence has somewhat refuted this suggestion [20,35]. Specifically, a number of research studies in golf have utilised flexibility or mobility assessment methods and shown that they appear to hold limited ecological validity in the sport. For example, both Donahue et al. [37] and Loock et al. [38] assessed flexibility using the sit-and-reach protocol, which seems both questionable as a method and a poor representation of any movement patterns golfers experience. More recently, Brennan et al. [20] determined the association between golf shot data and a seated thoracic rotation test in high-level male players, which has been suggested as a better method to assess mobility or flexibility for golfers [7]. Associations ranged from small to large with the same golf shot measures as the present study. However, this did not seem to hold true in the present female sample. It should be acknowledged though that flexibility and mobility may be very bespoke qualities that should be assessed on an individual basis. In contrast, it becomes hard to argue against the narrative that golfers will benefit most from improving their maximal and explosive force production abilities, given the findings presented in the recent and aforementioned meta-analysis [35].

### 4.2. Median Split Analysis

Figure 2, Figure 3 and Figure 4 provide the results for the median split analysis conducted for the force at 200 ms during the IMTP (Figure 2), force at 100 ms for the isometric bench press (Figure 3), and positive impulse during the CMJ (Figure 4). During the IMTP, players who exhibited a greater force at 200 ms also showed a significantly greater CHS (*g* = 1.13), ball speed (*g* = 0.90), and carry distance (*g* = 1.01), corroborating the importance of rapid force production in the lower body. This was further supported by the CMJ median split data, where players who produced a greater impulse also exhibited significantly greater ball speed (*g* = 0.93), carry distance (*g* = 1.29), and smash factor values (*g* = 1.27). Collectively, explosive force production in the lower body appears to be critical for amateur female golfers. In contrast, no significant differences were evident between groups when the sample was split for the isometric bench press. This is surprising given this particular metric showed the strongest association with CHS. Whilst challenging to fully explain, inspection of the individual data points in Figure 3 reveals that a few players with the fastest CHS and ball speeds reside in the lower-performing group. Thus, although recent evidence has shown that both maximal and explosive upper body force production are important for CHS in male players [35], it might be a physical quality that needs to be assessed on a more individual basis in female players.

### 4.3. Limitations

Despite the novel methods and findings in female golfers, a couple of limitations should be acknowledged in the present study. First, this investigation represents data over a single time point, which somewhat raises the question as to the repeatability of our findings. Thus, where possible, future research examining the link between physical attributes and female golf should aim to assess the seasonal variation in these characteristics or undertake a training intervention that may provide a more meaningful understanding of the interaction between physical performance and golf proxy measures. Second, our sample size was relatively small; thus, future research should, where possible, aim to recruit a larger population of female golfers to better elucidate the relationship between physical attributes and golf shot data. Related to this as well, despite the high level of amateur player in the present study, there is no guarantee that these data will transfer over to professional female golfers—a population for which there is a distinct lack of empirical evidence to date.

## 5. Conclusions

In summary, these findings demonstrate that CHS is the measure of golf performance that most commonly demonstrates large associations with physical performance data. From a physical testing perspective, rapid force production metrics seem to exhibit stronger relationships than peak force during isometric strength assessments, and the positive impulse (in line with prior research) appears to be a useful proxy measure from the CMJ test. Collectively, these data highlight that female golfers who demonstrate better explosive force production qualities are likely to exhibit faster CHS, greater ball speeds, and longer carry distances. In turn, these improved physical capacities may have the ability to positively affect competition-related metrics, such as strokes gained. Therefore, it is our suggestion that female golfers engage in both maximal strength and explosive strength training for potential enhancements to both their golf game and over-arching health.

## Figures and Tables

**Table 1:** Mean ± standard deviation (SD) data for driver performance and physical attributes, with intraclass correlation coefficients (ICC) with 95% confidence intervals (CI) and the standard error of the measurement (SEM).

Test/Metric	Mean ± SD	ICC (95% CI)	SEM
*Golf shot performance* (*driver*):			
Clubhead speed (mph)	94.34 ± 5.49	0.94 (0.88, 0.97)	1.34
Ball speed (mph)	134.08 ± 11.57	0.88 (0.77, 0.94)	4.01
Carry distance (yards)	198.02 ± 24.92	0.75 (0.52, 0.89)	12.46
Smash factor	1.42 ± 0.07	0.70 (0.49, 0.84)	0.04
*Anthropometry*:			
Body mass (kg)	64.04 ± 11.27	–	–
Height (cm)	166.26 ± 3.62	–	–
Wingspan (cm)	146.53 ± 15.59	–	–
*Isometric mid-thigh pull*:			
Peak force (N)	1062.46 ± 194.15	0.88 (0.78, 0.94)	67.26
Force @ 100 ms (N)	317.32 ± 119.60	0.63 (0.40, 0.80)	72.75
Force @ 200 ms (N)	595.17 ± 144.62	0.69 (0.48, 0.84)	80.52
Force @ 300 ms (N)	752.27 ± 143.60	0.77 (0.59, 0.89)	68.87
*Isometric bench press*:			
Peak force (N)	294.29 ± 83.38	0.92 (0.84, 0.96)	23.58
Force @ 100 ms (N)	149.86 ± 63.36	0.80 (0.64, 0.90)	28.34
Force @ 200 ms (N)	218.98 ± 77.77	0.89 (0.80, 0.95)	25.79
Force @ 300 ms (N)	266.54 ± 71.68	0.80 (0.64, 0.90)	32.06
*Countermovement jump*:			
Jump height (m)	0.24 ± 0.06	0.98 (0.96, 0.99)	0.001
Peak propulsive force (N)	1441.14 ± 253.02	0.95 (0.90, 0.98)	56.58
Peak propulsive power (W)	2509.89 ± 369.93	0.97 (0.93, 0.98)	64.07
Positive impulse (N·s)	472.39 ± 108.57	0.99 (0.98, 1.00)	10.86
*Medicine ball throw*:			
Distance (m)	3.18 ± 0.38	0.80 (0.64, 0.90)	0.17
*Mobility*:			
Seated thoracic rotation – left (°) Seated thoracic rotation – right (°)	61.33 ± 9.01 52.25 ± 12.49	0.78 (0.61, 0.89) 0.92 (0.85, 0.96)	4.23 3.53

mph = miles per hour; kg = kilograms; cm = centimetres; N = Newtons; m = metres; W = Watts; N·s = Newton seconds; ° = degrees.

## Data Availability

Data can be made available by contacting the corresponding author.
