# Correction: Robinson et al. Relationships and Within-Group Differences in Physical Attributes and Golf Performance in Elite Amateur Female Players. *Life* 2024, *14*, 674

**DOI:** 10.3390/life14091186

**Published:** 2024-09-20

**Authors:** Luke Robinson, Andrew Murray, Daniel Coughlan, Margo Mountjoy, Jack Wells, Rebecca Hembrough, Danny Glover, Fiona Scott, Anthony Turner, Chris Bishop

**Affiliations:** 1 London Sport Institute, Middlesex University, StoneX Stadium, Greenlands Lane, London NW4 1RL, UK; luker470@gmail.com (L.R.); a.n.turner@mdx.ac.uk (A.T.); 2 The R&A, St. Andrews KY16 9JD, UK; amurray@europeantourgroup.com (A.M.); dan@dancoughlan.com (D.C.); 3 European Tour Group, Wentworth Drive, Virginia Water, Surrey GU25 4LX, UK; 4 Ladies European Tour, Buckinghamshire Golf Club, Denham Court Drive, Uxbridge UB9 5PG, UK; dannyglover@nhs.net (D.G.); tourexercise@ladieseuropeantour.com (F.S.); 5 England Golf, National Golf Centre, The Broadway, Woodhall Spa LN10 6PU, UK; jack.wells@aru.ac.uk (J.W.); rebecca.hembrough@englandgolf.org (R.H.); 6 International Golf Federation, Maison du Sport International, Av. de Rhodanie 54, 1007 Lausanne, Switzerland; mountjm@mcmaster.ca; 7 Department of Sport and Exercise Sciences, Anglia Ruskin University, Cambridge CB1 1PT, UK

Addition of an Author

Jack Wells was not included as an author in the original publication [1].

Additional Affiliation(s)

In addition to affiliations 1–6, the updated affiliations should include the Department of Sport and Exercise Sciences at Anglia Ruskin University for Jack Wells. 

The corrected Author Contributions statement appears here.

**Author Contributions:** Conceptualization, L.R., A.M., D.C., M.M., J.W., R.H., D.G., F.S. and C.B.; Methodology, D.C., R.H. and C.B.; Formal analysis, L.R. and C.B.; Investigation, L.R. and F.S.; Data curation, D.C., F.S. and C.B.; Writing—original draft, L.R.; Writing—review & editing, A.M., D.C., M.M., J.W., R.H., D.G., F.S., A.T. and C.B.; Supervision, A.M., D.C., A.T. and C.B.; Project administration, J.W., D.G. and A.T. All authors have read and agreed to the published version of the manuscript.

The authors state that the scientific conclusions are unaffected. This correction was approved by the Academic Editor. The original publication has also been updated.
